# Genetic dissection of mammalian ERAD through comparative haploid and CRISPR forward genetic screens

**DOI:** 10.1038/ncomms11786

**Published:** 2016-06-10

**Authors:** Richard T. Timms, Sam A. Menzies, Iva A. Tchasovnikarova, Lea C. Christensen, James C. Williamson, Robin Antrobus, Gordon Dougan, Lars Ellgaard, Paul J. Lehner

**Affiliations:** 1 Department of Medicine, Cambridge Institute for Medical Research, Cambridge Biomedical Campus, Hills Road, Cambridge CB2 0XY, UK; 2 Department of Biology, University of Copenhagen, 2200 Copenhagen N, Denmark; 3 Wellcome Trust Sanger Institute, Wellcome Trust Genome Campus, Cambridge CB10 1SA, UK

## Abstract

The application of forward genetic screens to cultured human cells represents a powerful method to study gene function. The repurposing of the bacterial CRISPR/Cas9 system provides an effective method to disrupt gene function in mammalian cells, and has been applied to genome-wide screens. Here, we compare the efficacy of genome-wide CRISPR/Cas9-mediated forward genetic screens versus gene-trap mutagenesis screens in haploid human cells, which represent the existing ‘gold standard' method. This head-to-head comparison aimed to identify genes required for the endoplasmic reticulum-associated degradation (ERAD) of MHC class I molecules. The two approaches show high concordance (>70%), successfully identifying the majority of the known components of the canonical glycoprotein ERAD pathway. Both screens also identify a role for the uncharacterized gene *TXNDC11*, which we show encodes an EDEM2/3-associated disulphide reductase. Genome-wide CRISPR/Cas9-mediated screens together with haploid genetic screens provide a powerful addition to the forward genetic toolbox.

## Abstract (web summary)

CRISPR/Cas9-mediated forward genetic screens and gene-trap mutagenesis screens in haploid cells are both powerful techniques to examine gene function. Here, the authors show the two approaches have high concordance and identify an uncharacterized gene, *TXNDC11*, which is involved in endoplasmic reticulum-associated degradation.

## Graphical abstract

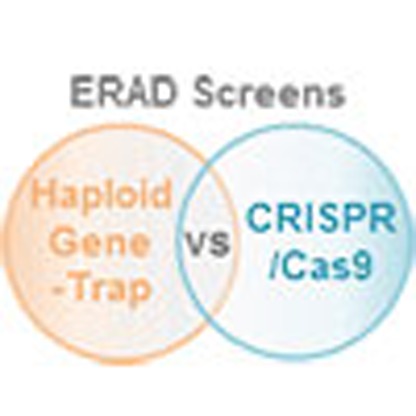

Although our understanding of the gene has advanced considerably since the pioneering work of Muller in the 1920s (ref. 1), the basic principle of the forward genetic screen has remained the same: a population of cells are mutagenized to create a library of gene knockouts, which can then be screened for mutants defective in the pathway of interest and the responsible genes identified by mapping the causative mutations. This approach has proved enormously successful in identifying gene function in a range of model eukaryotic organisms, but the difficulty in generating bi-allelic mutations in diploid cells has limited the application of this approach in human cells.

Until recently the only practical way of carrying out genome-wide loss-of-function screens in cultured human cells was via RNA interference, whereby short double-stranded RNAs loaded onto the RNA-induced silencing complex target the endonucleolytic cleavage of cognate messenger RNA (mRNA) targets^2^. This method circumvents the problem of diploidy as it acts at the mRNA level, and can therefore be carried out in the cell type of choice. Although genome-wide screens performed via transfection of short interfering RNAs (siRNAs) are expensive and labour-intensive, pooled screens can be performed using lentiviral expression libraries of short hairpin RNA (shRNA) constructs^3^,^4^. However, RNA interference-based screening approaches are limited by: (1) incomplete knockdown of gene expression, (2) the variable degree of suppression observed with different siRNAs and (3) off-target effects, whereby a non-cognate mRNA is silenced to a similar extent as the intended target^5^.

An important breakthrough in the field of experimental human genetics was the demonstration that the near-haploid human KBM7 cell line could be used to perform forward genetic screens^6^. Gene inactivation on a haploid background results in a loss-of-gene function, and so insertional mutagenesis of KBM7 cells with a gene-trap retroviral vector can be used to create a library of gene knockouts^7^. Haploid genetic screens in KBM7 cells and their partially reprogrammed derivative HAP1 have successfully assigned functions to a suite of genes involved in a wide variety of cellular processes^8^,^9^,^10^,^11^, and the derivation of haploid embryonic stem cells^12^,^13^ now permits a similar approach in cultured murine cells^14^,^15^. As such, haploid genetic screens currently represent the ‘best-in-class' method for the forward genetic analysis of cultured human cells.

Custom genome editing using programmable nucleases is a transformative technology for cell biologists. The deployment of the bacterial CRISPR/Cas9 system in human cells now offers—for the first time—a practical method to generate bi-allelic mutation and hence functional gene knockouts^16^. Through the synthesis of a large library of guide RNAs (sgRNAs) to target the Cas9 nuclease to all known genes, CRISPR-mediated gene disruption can be applied on a genome-scale to perform forward genetic screens^17^,^18^,^19^. The major attraction of this approach is that, unlike haploid screens, CRISPR screens can theoretically be performed in any cell type, including primary cells^20^ or even *in vivo*^21^. However, there are some potential technical caveats that may limit the effectiveness of CRISPR screens, such as the variable efficacy of gene disruption by different sgRNAs, the potential for off-target effects, and the requirement for maintaining even representation across a large expression library containing thousands of individual sgRNAs.

Here, we set out to directly compare the effectiveness of the two approaches in a head-to-head comparison. We chose to study the process of endoplasmic reticulum-associated degradation (ERAD), in which misfolded proteins of the early secretory pathway are recruited to the cellular dislocation machinery and retrotranslocated back across the ER membrane to the cytosol for proteasomal degradation^22^. Previously, we showed that the major histocompatibility complex class I (MHC-I) heavy chain HLA-A2 is a substrate for ERAD through the canonical glycoprotein quality control pathway^23^,^24^. This pathway is initiated by the sequential trimming of mannose residues from the N-linked glycans of misfolded polypeptides by the EDEM proteins^25^, which mediates their targeting to the Hrd1–SEL1L complex^26^. These misfolded substrates are subsequently retrotranslocated from the ER, ubiquitinated, released into the cytosol and finally degraded by the proteasome.

Our results indicate that both haploid gene-trap and CRISPR/Cas9-mediated screening techniques represent highly effective tools to identify the genes involved in cellular processes. Moreover, both approaches highlight an essential role for the uncharacterized gene *TXNDC11* in glycoprotein ERAD, which we show encodes an EDEM2/3-associated disulphide reductase.

## Results

### A fluorescent reporter system to monitor MHC-I ERAD

**Figure 1:**
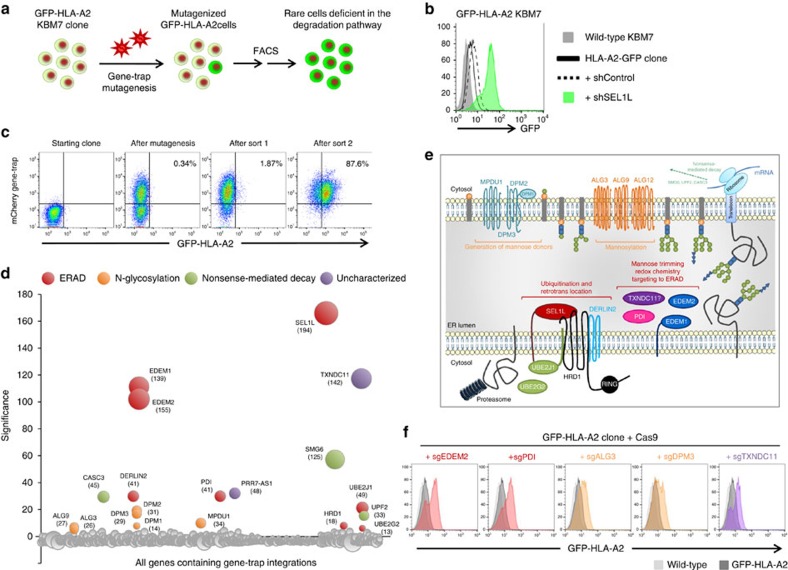
A haploid genetic screen identifies genes required for glycoprotein ERAD. (**a**) Schematic view of the haploid genetic screen to identify the genes required for glycoprotein ERAD. (**b**) Isolation of a KBM7 clone in which the GFP-HLA-A2 reporter is undergoing ERAD, as evidenced by increased GFP-HLA-A2 expression upon depletion of SEL1L. (**c**) Following gene-trap retroviral mutagenesis, rare GFP^high^ cells were isolated via two rounds of FACS. (**d**) Bubble plot illustrating the hits from the screen. Bubble size is proportional to the number of independent gene-trap integrations in each gene predicted to inactivate gene expression (number in parentheses). (**e**) Schematic representation of the cellular pathways in which the screen hits function. (**f**) CRISPR/Cas9-mediated validation of a subset of the screen hits.

To perform parallel haploid and CRISPR forward genetic screens to identify the genes involved in MHC-I ERAD (Fig. 1a), we first needed to establish an ERAD reporter system in near-haploid KBM7 cells^11^,^27^. We transduced KBM7 cells with a lentiviral vector encoding a green fluorescent protein (GFP)-tagged HLA-A2 and isolated single cell clones. For the screens we selected a clone that exhibited a low level of GFP fluorescence at steady-state and which showed a robust increase in GFP fluorescence upon depletion of SEL1L (Fig. 1b), a key component of the MHC-I ERAD pathway^23^, indicating that the GFP-HLA-A2 fusion protein was indeed degraded via ERAD.

### A haploid gene-trap screen for MHC-I ERAD

Following retroviral gene-trap mutagenesis we sequentially enriched for GFP^high^ cells by two rounds of fluorescence-activated cell sorting (FACS), and isolated a relatively pure population of GFP^high^ cells (Fig. 1c). To identify the inactivated genes responsible for the impaired degradation of GFP-HLA-A2, we mapped the retroviral integration sites in the selected cells by linear amplification-mediated PCR (LAM-PCR) and Illumina sequencing^7^. This revealed a suite of genes that were significantly enriched in the selected cells as compared with an unselected control population (Fig. 1d and Supplementary Data 1). These genes could be readily grouped into three functional classes. The largest group comprised genes known to be involved in ERAD, including the mannosidases EDEM1 and EDEM2, the E3 ubiquitin ligase Hrd1 (encoded by the *SYVN1* gene) and its binding partners SEL1L and Derlin-2, and the E2 ubiquitin conjugases UBE2J1 and UBE2G2 (Fig. 1e). We have previously shown a functional requirement for the majority of these genes in the MHC-I ERAD pathway^23^,^24^. The second group included a large number of genes involved in N-glycosylation, including all three members of the dolichol–phosphate–mannose (DPM) synthase complex required for the generation of mannosyl donors^28^, and two mannosyltransferase enzymes, ALG3 and ALG9 (Fig. 1e). As the glycoprotein ERAD machinery recognizes substrates through trimming of mannose residues on N-glycans^25^, it follows that deletion of any of these genes might prevent recognition of GFP-HLA-A2 as an ERAD substrate. Unexpectedly we also identified three genes known to be involved in nonsense-mediated decay, suggesting that the GFP reporter was also subject to degradation at the RNA level. We selected a subset of the genes for further validation, and found that CRISPR/Cas9-mediated gene disruption did indeed result in impaired degradation of the GFP-HLA-A2 substrate (Fig. 1f).

### A parallel CRISPR screen for MHC-I ERAD

**Figure 2:**
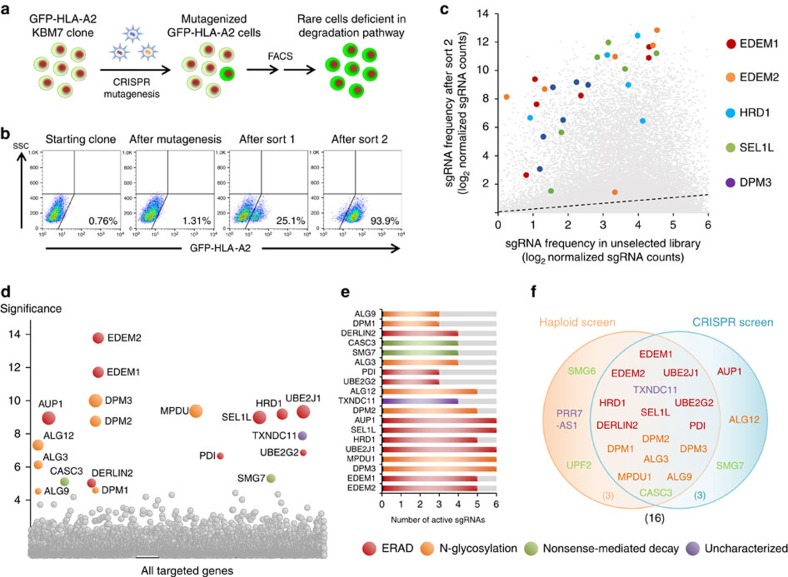
A parallel CRISPR/Cas9-mediated forward genetic screen identifies genes required for glycoprotein ERAD. (**a**,**b**) A CRISPR-mediated forward genetic screen to identify the genes required for glycoprotein ERAD. Cas9 was stably expressed in the same GFP-HLA-A2 KBM7 clone, CRISPR-mediated mutagenesis was performed using the GeCKO v2 sgRNA library, and rare GFP^high^ cells isolated by two rounds of FACS. (**c**) Individual sgRNAs targeting ERAD factors previously identified by the haploid genetic screen were highly enriched in the selected GFP^high^ cells as compared with the unselected mutant library. The dotted line represents the linear regression line of best fit. (**d**) Bubble plot illustrating the hits from the CRISPR screen. The RSA algorithm was used to identify the significantly enriched genes targeted in the selected cells. Bubble size is proportional to the number of active sgRNAs per gene. (**e**) The number of active sgRNAs for each of the screen hits, as identified by the RSA algorithm. (**f**) Overlap of the screen hits from the haploid and CRISPR forward genetic screens.

Forward genetic screens using genome-wide CRISPR/Cas9-mediated gene disruption have recently emerged as an alternative method to gene-trap haploid screens to examine gene function in cultured mammalian cells^17^,^18^,^19^,^29^. To directly compare the efficacy of the two techniques, we performed a parallel CRISPR screen in the same KBM7 clone carrying the GFP-HLA-A2 reporter (Fig. 2a). We stably introduced the Cas9 nuclease via lentiviral transduction and then carried out genome-wide CRISPR-mediated mutagenesis using the GeCKO v2 library, which contains 123,411 sgRNAs targeting 19,050 protein-coding genes^30^. Again, we enriched for mutant GFP^high^ cells by two round of FACS (Fig. 2b), and then quantified sgRNA abundance in the selected cells versus the unsorted mutant library by deep sequencing^18^. We observed strong enrichment for individual sgRNAs targeting the ERAD factors identified by the haploid screen (Fig. 2c). We then used the RSA algorithm^31^ to identify the hits that were consistently enriched by multiple sgRNAs targeting the same gene (Fig. 2d,e and Supplementary Data 2). Strikingly, high concordance was observed between the data from the CRISPR screen and the haploid screen. Altogether, the two approaches identified 22 significantly enriched genes: 16 of these genes (>70%) were identified in both screens, while 3 additional genes were differentially highlighted by each technique (Fig. 2f).

In an attempt to further understand the reasons for this differential identification, we selected four of these genes for further validation (Supplementary Fig. 1). We found that all four represented true hits: in every case, individual CRISPR/Cas9-mediated gene disruption experiments resulted in an increase in GFP-HLA-A2 levels (Supplementary Fig. 1a). *SMG6* and *UPF2* therefore represent false-negatives on the CRISPR screen. Examination of the unselected control library showed that all six sgRNAs targeting these two genes were detected in this sample, but that only one of the six sgRNAs in each case was enriched in the selected population (Supplementary Fig. 1b). This false-negative result is therefore likely to be due to these guides being ineffective. Similarly, *SMG7* and *ALG12* represent false-negatives on the haploid gene-trap screen. Gene-trap integrations into *SMG7* were not enriched in the selected cells (Supplementary Fig. 1c). *ALG12* falls just below our stringent cut-off for statistical significance, suggesting that this gene would have been identified had more unique integrations sites been mapped, which could be achieved through deeper sequencing coverage. A relatively small number of integrations into *ALG12* were detected, suggesting that this gene may lie in a relative ‘cold-spot' for retroviral integration (Supplementary Fig. 1c). Indeed, one potential advantage of the CRISPR approach is that it is not subject to the inherent bias of insertional retroviral mutagenesis. In our comparison the sgRNA library provided more even mutagenic coverage across the genome (Supplementary Fig. 1d,e), although the retroviral gene-trap vector does preferentially target transcriptionally-active genes^7^. Overall, these data suggest that the two methods are both highly effective at identifying relevant genes, although neither approach alone would appear to be saturating.

### TXNDC11 is an ER-resident thioredoxin domain protein

**Figure 3:**
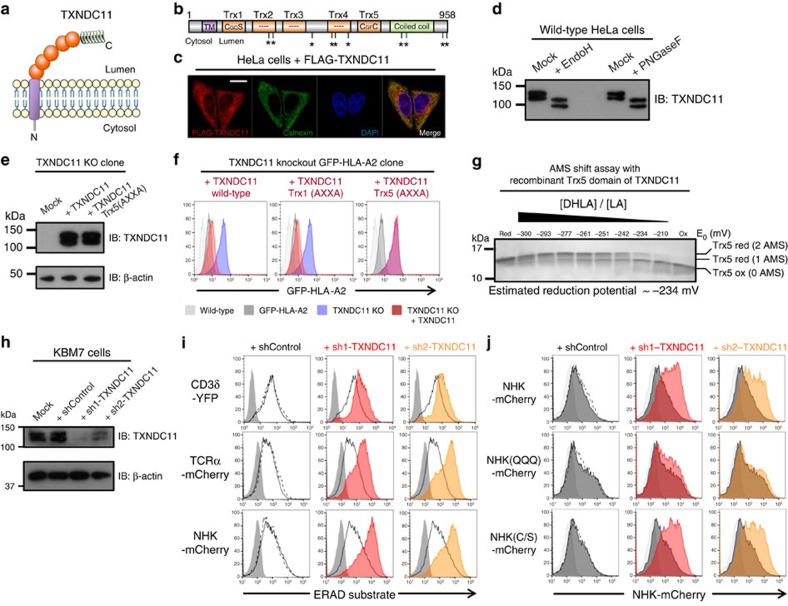
TXNDC11 is a disulphide reductase involved in glycoprotein ERAD. (**a**,**b**) Predicted architecture of the TXNDC11 protein. In (**b**), potential N-glycosylation sites are indicated with asterisks. (**c**,**d**) Epitope-tagged TXNDC11 predominantly localizes to the ER as assessed by co-localization with calnexin by immunofluorescence (**c**), supported biochemically by sensitivity to digestion with EndoH (**d**). (**c**) Scale bar, 20 μm. (**e**,**f**) Genetic reconstitution of TXNDC11 knockout cells. Expression of wild-type TXNDC11 rescues GFP-HLA-A2 degradation in TXNDC11 knockout cells. Mutation of the active site cysteine residues of the Trx5 domain, but not the Trx1 domain, abolishes the function of TXNDC11. (**g**) Redox titration of the TXNDC11 Trx5 domain with lipoic acid (DHLA/LA). The redox state of the purified Trx5 domain was assessed using an AMS shift assay in the presence of increasing ratios of DHLA/LA. Controls of the reduced (10 mM TCEP, left lane) and oxidized (untreated protein, right lane) forms of the protein are included. See Supplementary Fig. 6 for further discussion. (**h**–**j**) Depletion of TXNDC11 impairs the degradation of model glycoprotein ERAD substrates. (**h**) Immunoblot validation of efficient shRNA-mediated depletion of TXNDC11 in KBM7 cells. (**i**) KBM7 cell lines stably expressing fluorescently tagged CD3d (top panel), TCRa (middle panel) and NHK (bottom panel) were transduced with shRNA expression vectors targeting TXNDC11, and protein levels of the ERAD substrates measured by flow cytometry. (**j**) Depletion of TXNDC11 impairs the degradation of a NHK mutant lacking any cysteine residues. KBM7 cell lines were established stably expressing mCherry-tagged NHK (top panel), NHK(QQQ), which cannot be *N*-glycosylated (middle panel) or NHK(C/S), which lacks cysteine residues (bottom panel). The three cell lines were transduced with shRNA expression vectors targeting TXNDC11, and stabilization of NHK protein levels was measured by flow cytometry. Depletion of TXNDC11 impairs the ERAD of the NHK(C/S) mutant to a similar extent to that of wild-type NHK, but does not have a significant effect on the degradation of the non-glycosylated NHK(QQQ) variant.

Forward genetic screens provide a powerful means to identify functional roles for novel genes in cellular pathways. Both the haploid and CRISPR screening methods identified TXNDC11, an uncharacterized member of the protein disulphide isomerase (PDI) family, as a novel protein putatively involved in ERAD. TXNDC11, also known as EFP1, was originally identified as a binding partner for the hydrogen peroxide-generating enzyme dual oxidase 1 (DUOX1) in a yeast two-hybrid screen^32^, but nothing is known about its cellular function. TXNDC11 is annotated as encoding a transmembrane protein with a short cytoplasmic amino-terminus, a single transmembrane domain and a large luminal portion (Fig. 3a). Homology modelling of the luminal portion predicted five thioredoxin-like (Trx) domains at the core of the protein, plus a coiled-coil region at the carboxy-terminus (Fig. 3b and Supplementary Fig. 2). Such coiled-coil regions often mediate protein oligomerization, and indeed we observed oligomerization of recombinant TXNDC11 coiled-coil domain constructs with different affinity tags expressed in *Escherichia coli* (Supplementary Fig. 3). However, the coiled-coil domain was not critical for TXNDC11 function, as exogenous expression of a TXNDC11 mutant construct lacking this domain was still able to rescue the degradation of GFP-HLA-A2 in TXNDC11 knockout cells generated through CRISPR/Cas9-mediated gene disruption (Supplementary Fig. 4). Immunofluorescence analysis of epitope-tagged TXNDC11 in HeLa cells suggested that the protein resides predominantly in the ER (Fig. 3c), and this was supported biochemically by sensitivity to digestion with Endoglycosidase H (EndoH) (Fig. 3d). Both endogenous (Fig. 3d) and exogenous (Fig. 3e) TXNDC11 migrated as a doublet on polyacrylamide gels; the faster migrating band may represent a soluble TXNDC11 isoform derived from partial signal peptidase cleavage (Supplementary Fig. 5).

### Redox activity of TXNDC11 is required for efficient MHC-I ERAD

PDI-family proteins catalyze the formation and reduction of disulphide bonds in the ER through catalytic thioredoxin-like domains containing two cysteine residues in a CXXC active site motif. Of the five predicted Trx domains in TXNDC11, only Trx5 contains a CXXC active site motif; Trx1 contains a CXXS motif, while Trx2, Trx3 and Trx4 lack any active site cysteine residues (Fig. 3b). To determine whether oxidoreductase activity of TXNDC11 might be important for its function, we therefore mutated the active site cysteine residues of Trx5 to alanine (Fig. 3e). Unlike the wild-type TXNDC11 protein, the TXNDC11 Trx5 (AXXA) mutant was no longer able to rescue GFP-HLA-A2 degradation in cells lacking TXNDC11, supporting a redox role for TXNDC11 (Fig. 3f). We therefore expressed the active Trx5 domain in *E. coli* (Supplementary Fig. 6) and examined the properties of the purified domain *in vitro*. The active site disulphide of Trx5 was found to be very stable, with an estimated reduction potential of ∼−234 mV (Fig. 3g and Supplementary Fig. 5d). This value is considerably more reducing than that of other PDI-family members^33^,^34^,^35^, indicating a redox function for TXNDC11 as a reductase.

The relevant target of this reductase activity could either be the ERAD substrate itself or a component of the ERAD machinery. We found that depletion of TXNDC11 (Fig. 3h) also impaired the degradation of three additional ERAD substrates: the unassembled CD3 delta chain (CD3δ), the unassembled TCR alpha chain (TCRα) and the Null-Hong Kong (NHK) variant of alpha-1 antitrypsin (Fig. 3i). We further explored substrate requirements by constructing two mutants of NHK: NHK(QQQ), in which all N-glycosylated asparagine residues are mutated to glutamine^36^, and NHK(C/S), in which the single cysteine residue in NHK (through which it forms aberrant disulphide-bonded dimers^37^) is mutated to serine (Fig. 3j). Consistent with the notion that TXNDC11 acts in the glycoprotein ERAD pathway, shRNA-mediated knockdown of TXNDC11 did not have a significant effect on the stability of the non-glycosylated NHK(QQQ) variant (Fig. 3j). However, TXNDC11 depletion impaired the degradation of the NHK(C/S) mutant similarly to wild-type NHK (Fig. 3j). This demonstration that TXNDC11 is required for the efficient ERAD of a substrate that lacks any cysteine residues supports the idea that the relevant target of the TXNDC11 reductase activity may not be the ERAD substrate, but rather another component of the ERAD machinery itself.

### TXNDC11 binds known members of the ERAD machinery

**Figure 4:**
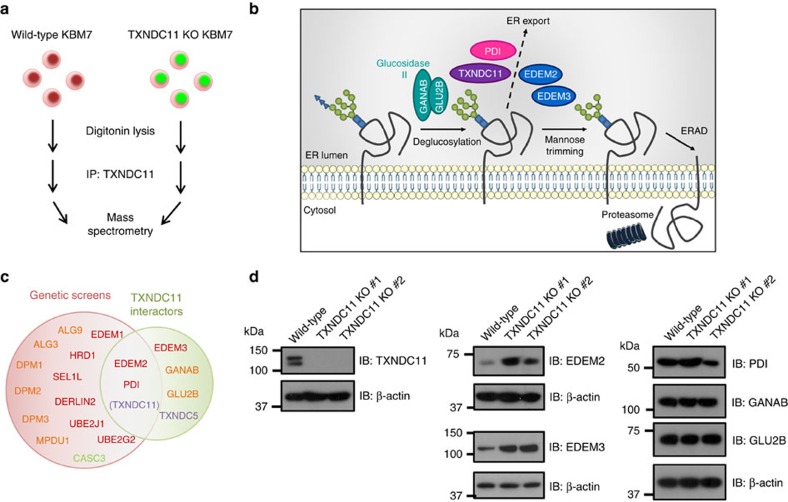
TXNDC11 interacts with EDEM2/3 and the alpha-glucosidase complex. (**a**) Overview of the experiment designed to identify TXNDC11-interacting partners by MS. (**b**) Schematic representation of the role of TXNDC11 and its binding partners in the ERAD pathway. (**c**) Overlap between the hits from the genetic screens with the TXNDC11 binding partners identified by MS. (**d**) Loss of TXNDC11 increases the protein levels of EDEM2 and EDEM3. The protein levels of the TXNDC11-interacting partners were analyzed by immunoblot in two independent TXNDC11-deficient clones.

To gain further insight into the cellular role of TXNDC11, we sought to identify TXNDC11 binding partners. Immunoprecipitation of endogenous TXNDC11 from wild-type cells followed by mass spectrometry (MS) (Fig. 4a) identified six putative interacting proteins that were absent from control samples: the mannosidases EDEM2 and EDEM3, the oxidoreductases PDI (encoded by the *P4HB* gene) and TXNDC5, and both subunits of the alpha-glucosidase complex^38^ (encoded by *GANAB* and *PRKCSH*) (Table 1, Supplementary Fig. 7 and Supplementary Data 3). These results firmly implicate TXNDC11 in glycoprotein quality control, at the intersection of those pathways that regulate protein folding, export from the ER, and targeting for ERAD (Fig. 4b). The overlap between this proteomic dataset and our data from the genetic screens indicated that the interactions between TXNDC11 and EDEM2 and PDI were likely to be critical for its role in ERAD (Fig. 4c), and knockout of TXNDC11 resulted in increased levels of EDEM2 and EDEM3 (Fig. 4d). Finally we found that TXNDC11 transcript levels were upregulated in response to ER stress (Supplementary Fig. 8a), which could potentially be mediated through a canonical ATF6 binding site^39^ in the TXNDC11 promoter (Supplementary Fig. 8b). Taken together, these data suggest a model whereby the redox activity of TXNDC11, acting in concert with EDEM proteins and PDI, is required for glycoprotein ERAD.

## Discussion

The aim of this study was to compare the effectiveness of genome-wide CRISPR/Cas9-mediated forward genetic screens with the ‘gold standard' gene-trap haploid genetic screens at identifying genes required for ERAD. We find that both screening modalities represent highly effective approaches to identify the genes involved in the cellular pathways. A similar conclusion was recently reached independently by Wang *et al*.,^40^ who showed that, with the exception of the diploid chromosome 8, gene-trap and CRISPR-mediated mutagenesis produced highly concordant results in defining the essential genes of KBM7 cells.

Our data demonstrate that FACS-based forward genetic screens using fluorescently tagged substrates represent an effective approach to study the genes required for ERAD. The genes identified by the haploid and CRISPR screens agree well with our previous work using siRNA screening and candidate gene approaches to define the genes involved in MHC-I ERAD. However, even the combination of hits from the two genetic screens is unable to reach saturation. Our previous work demonstrated a requirement for the p97 ATPase, its cofactor Ufd1 and the ER lectin XTP3-B in MHC-I ERAD^24^, but these components were not identified in either screen, possibly because their depletion delays cell growth, or in the case of p97 components is lethal. We previously identified Hrd1 as the E3 ubiquitin ligase and UBE2J1 as the E2 ubiquitin conjugase involved, together with a role for SEL1L and EDEM1 (refs 23, 24). The data from the genetic screens described here validate a role for these factors, identify a role for additional genes known to be involved in glycoprotein ERAD, and implicate *TXNDC11* as a novel gene required for MHC-I ERAD. TXNDC11 joins a growing list of PDI-family proteins known to be involved in ER homoeostasis^41^. In particular, there are intriguing parallels between TXNDC11 and ERdj5, another ER-resident protein that contains thioredoxin-like domains with highly reducing active sites^33^,^37^. ERdj5 was originally identified as an interacting partner of EDEM1 (ref. 37), while here we show that TXNDC11 binds to both EDEM2 and EDEM3. ERdj5 is thought to accelerate ERAD by reducing disulphide bonds in the ERAD substrate itself, before retrotranslocation^37^. Our data suggest that the relevant target of TXNDC11 activity may not be the ERAD substrate itself, as the cysteine-less substrate NHK(C/S) was also sensitive to loss of TXNDC11, but rather a component of the ERAD machinery (Supplementary Fig. 8).

There are advantages and disadvantages associated with each genetic screening approach. From a practical standpoint, retroviral gene-trap mutagenesis is more straightforward as it does not require the prior construction and propagation of a large sgRNA library. The analysis of the screen by next-generation sequencing is considerably easier for a CRISPR screen, however, requiring only a routine PCR reaction to amplify the sgRNA sequences; a more involved inverse PCR^6^ or LAM-PCR^7^ reaction is required to amplify the genomic DNA flanking retroviral gene-trap integration sites. Another potential advantage of a retroviral mutagen is that it will target all accessible chromatin, and therefore has the capacity to identify unannotated genes and regulatory genetic elements. Although it was not investigated further, our haploid screen identified the antisense RNA encoded by *PRR7-AS1* as a putative hit, which is an example of a locus not included in the GeCKO v2 sgRNA library. The CRISPR/Cas9-mediated mutagenesis appeared to be more efficient in our comparison, however, leading to a greater proportion of GFP^high^ cells after the initial FACS enrichment. However, as we carried out the CRISPR screen in a haploid KBM7 clone, it is unclear from these data how far this efficiency might drop in diploid cells. The real benefits of the CRISPR approach lie in its general applicability to non-haploid cells, including diploid cell lines and primary cells. The modular nature of the CRISPR system also makes it potentially far more versatile than gene-trap mutagenesis. For example, CRISPR has already been adapted to produce activation of gene expression and perform genome-wide overexpression screens^42^,^43^. Ultimately the choice of cell type and mutagenesis method will depend on the particular design of the experiment, with the two methods providing complementary approaches to examine gene function.

## Methods

### Cell culture

KBM7 cells, obtained from Dr Brent Cochran^44^, and HEK 293ET cells, a generous gift from Dr Felix Randow, were cultured in IMDM plus 10% fetal calf serum (FCS) and penicillin/streptomycin. HeLa cells were obtained from ECACC and were grown in RPMI 1640 plus 10% FCS and penicillin/streptomycin.

### Antibodies

Primary antibodies used were as follows: rabbit α-TXNDC11 (Abcam, ab188329; 1:5,000), rabbit α-FLAG (Sigma-Aldrich, F7425; 1:10,000), mouse M1 α-FLAG (Sigma-Aldrich, F3040; 1:10,000), rabbit α-EDEM2 (Sigma-Aldrich, E1728; 1:5,000), rabbit α-EDEM3 (Sigma-Aldrich, E0409; 1:5,000), rabbit α-PDI (Cell Signaling, #2446; 1:5,000), rabbit α-GANAB (GeneTex, GTX102237; 1:5,000), rabbit α-SEL1L (Santa Cruz Biotechnology, sc-48080; 1:2,000), mouse α-calnexin (AF8, a kind gift from M. Brenner, Harvard Medical School; 1:10,000) and mouse α-β-actin (Sigma-Aldrich, A5316; 1:10,000).

### Lentiviral expression vectors

For CRISPR/Cas9-mediated gene disruption, oligonucleotides (Sigma-Aldrich) for top and bottom strands of the sgRNA were annealed, and then cloned into the lentiviral sgRNA expression vector pKLV-U6gRNA(BbsI)-PGKpuro2ABFP (Addgene #50946, kindly deposited by Dr Kosuke Yusa^19^). Lentiviral expression of shRNA constructs was achieved using the pHR-SIREN vector, with hairpins cloned in as BamHI–EcoRI fragments. All sequences are detailed in Supplementary Data 4. For the expression of exogenous genes, the vectors pHRSIN-P_SFFV_-GFP-P_PGK_-Hygro and pHRSIN-P_SFFV_-GFP-P_PGK_-Blasto were used, with the gene of interest inserted in place of GFP. Coding sequence for TXNDC11 (corresponding to Uniprot isoform 2, identifier Q6PKC3-2) that was sgRNA- and shRNA-resistant was ordered as a series of gBlocks (IDT) and assembled using the Gibson Assembly method (NEB). TXNDC11 mutants were created by site-directed mutagenesis using standard protocols. In all cases, lentivirus was produced by transfecting HEK 293ET cells with the lentiviral vector plus the packaging plasmids pCMVΔR8.91 and pMD.G using TransIT-293 reagent (Mirus) as recommended by the manufacturer. The viral supernatant was collected 48 h later, passed through a 0.45 μm filter and target cells transduced by spin infection at 700 × *g* for 60 min.

### Haploid genetic screen

The haploid genetic screen was carried out exactly as described^11^. Approximately 10^8^ GFP-HLA-A2 KBM7 cells were mutagenized with the gene-trap retrovirus Z-loxP-mCherry. The mutagenized cells were then grown for 7 days before the first sort to enrich for rare GFP^high^ cells; a second sort was performed to further purify the GFP^high^ population 10 days later. Genomic DNA was extracted (Puregene Core Kit A, Qiagen) from the resulting selected cells and an aliquot of the unsorted mutagenized library grown for the same amount of time. The retroviral gene-trap integration sites in both the control and selected populations were then mapped by LAM-PCR as described previously^11^. For the bubble plot presented in Fig. 1d, the degree of enrichment of each gene in the selected population compared to the unselected cells was calculated using a Bonferroni-corrected one-sided Fisher's exact test. All genes found to contain gene-trap integrations are listed alphabetically on the *x* axis; bubble size is proportional to the number of unique gene-trap integrations predicted to inactivate gene expression (given in parentheses).

### CRISPR screen

The Cas9 nuclease was stably expressed in GFP-HLA-A2 KBM7 cells by lentiviral transduction. Approximately 10^8^ cells were then transduced with the GeCKO v2 sgRNA library (Addgene cat#1000000047, kindly deposited by Prof. Feng Zhang^30^) at a multiplicity of infection of around 0.2. Untransduced cells were removed from the library through puromycin selection (0.75 μg ml^−1^) commencing 48 h after transduction. Rare GFP^high^ cells were then enriched by sequential rounds of FACS, with the first sort taking place 7 days after transduction with the sgRNA library and the second sort a further 10 days later. Genomic DNA was extracted (Puregene Core Kit A, Qiagen) from both the sorted cells and an unselected pool of mutagenized cells grown for the same amount of time. sgRNA sequences were amplified by two rounds of PCR, with the second round primers containing the necessary adaptors for Illumina sequencing (Supplementary Data 4). Sequencing was carried out using a 50 bp single-end read on an Illumina HiSeq2500 instrument using a custom primer binding immediately upstream of the 20 bp variable segment of the sgRNA. The 3′ end of the resulting reads were trimmed of the constant portion of the sgRNA, and then mapped to an index of all of the sgRNA sequences in the GeCKO v2 library using Bowtie 2. The resulting sgRNA count tables were then analyzed using the RSA^31^, MAGeCK^45^ and HiTSelect^46^ algorithms using the default settings. For the bubble plot presented in Fig. 2c, bubble size is proportional to the number of active sgRNAs per gene.

### Flow cytometry

Cells were washed once with PBS, fixed in 1% PFA and analyzed on a FACSCalibur (BD). For sorting, cells were resuspended in PBS+2% FCS and FACS was carried on an Influx cell sorter (BD).

### Immunofluorescence

HeLa cells were seeded on glass coverslips, fixed with 4% PFA, permeabilized with 0.5% Triton X-100, and then blocked for at least 30 min using 4% BSA dissolved in PBS+0.1% Tween-20 (PBS-T). Primary antibody (diluted in blocking solution) was then applied for 1 h, and following five washes in PBS-T, fluorophore-conjugated secondary antibody was applied for 45 min. Coverslips were mounted in Prolong anti-fade reagent with DAPI (Molecular Probes) and imaged using a Nikon LSM710 laser scanning confocal microscope (Zeiss). Images were processed using Adobe Photoshop (Adobe, CA) or GIMP 2.

### Plasmids for *E. coli* expression

Coding sequence to express the Trx5 domain of TXNDC11, codon-optimized for *E. coli*, was ordered as a gBlock (IDT) and cloned into the bacterial expression vector pET39b-Ub19 (a kind gift from Prof. V. Dötsch, Goethe University Frankfurt, Germany^47^) between the NcoI and BamHI restriction sites. To prevent the formation of disulphide-linked oligomers, a non-active site cysteine residue was mutated to serine (C743S). The resulting plasmid pLE524 encodes Ub-His-Trx5 C743S, containing N-terminal ubiquitin and His tags, a TEV protease cleavage site, a four residue linker (GAMG) followed by 123 residues of TXNDC11, encompassing the region from His^650^ to Asp^772^ (HLIGS…LHHSD). Two TXNDC11 Trx5-coiled-coil (CC) constructs were created, one in the pET39b-Ub19 vector as described above (Ub-His-Trx5-CC C743S/C788S encoded by pLE525) and the other in pMAL-c5x (NEB) (MBP-Trx5-CC C743S/C788S encoded by pLE523), cloned in between the NdeI and EcoRI sites (for an N-terminal maltose-binding protein tag). These constructs encompassed the region of TXNDC11 from His^650^ to the end of the protein, and contained two cysteine to serine mutations to prevent the formation of disulphide-linked oligomers (C743S and C788S).

### Expression and purification of TXNDC11 constructs containing the coiled-coil domain

Plasmids pLE523 and/or pLE525 were transformed into competent BL21(DE3) cells and plated onto LB plates containing the appropriate antibiotic(s) (ampicillin (100 μg ml^−1^) and/or kanamycin (50 μg m^−1^l)). The plates were grown overnight at 37 °C. One colony was inoculated, and grown overnight in 125 ml LB medium with appropriate antibiotic(s) at 37 °C. The overnight culture was diluted to an optical density at 600 nm (OD_600_) of 0.1 in LB medium containing antibiotic(s) and grown at 37 °C. At OD_600_=0.75 protein expression was induced with 1 mM isopropyl β-D-1-thiogalactopyranoside (IPTG), and the cells grown overnight at 20 °C. Cells were harvested by centrifugation, and cell pellets dissolved in 10 ml fusion protein lysis buffer (50 mM Tris, 25 mM NaCl, 5% glycerol, pH 7.8) with 1 mM phenylmethylsulfonyl fluoride (PMSF) and protease inhibitor cocktail mixture (Roche) per litre of starting culture. Lysozyme (Merck) was added to a final concentration of 1 mg ml^−1^ and the cell suspension was incubated for 1 h on ice. Finally, cells were broken open by sonication and then centrifuged for 1 h at 27,000 × *g* to generate the cleared lysate.

For Ni-NTA purification, the cleared lysate containing co-expressed MBP-Trx5-CC C743S/C788S and Ub-His-Trx5-CC C743S/C788S was filtered and diluted with an equal volume of Ni-NTA wash buffer (50 mM Tris, 20 mM imidazole, 250 mM NaCl, 1% glycerol, pH 7.8). This solution was then incubated on a rolling table for 1 h and 15 min at 4 °C with Ni-NTA beads (Qiagen) prewashed in Ni-NTA wash buffer. The beads were poured into an empty column and the flowthrough (FT) collected. Beads were then washed with 3 × 10 column volumes (CVs) of Ni-NTA wash buffer followed by elution of bound proteins with 9 CVs of Ni-NTA elution buffer (50 mM Tris, 400 mM imidazole, 250 mM NaCl, 1% glycerol pH 7.8). Selected fractions were separated on a 12% SDS–PAGE gel in reducing loading buffer unless otherwise stated.

For amylose resin purification, the FT from the Ni-NTA purification was diluted 10 times in amylose column buffer (20 mM Tris–HCl, 200 mM NaCl, pH 7.4) and incubated on a rolling table for 1 h and 15 min at 4 °C with amylose resin (New England Biolabs) prewashed in amylose column buffer. The resin was poured into an empty column and the FT collected. The resin was then washed with 5 × 4 CVs of amylose column buffer. Elution from the amylose resin was performed with 4 CVs of amylose elution buffer (20 mM Tris–HCl, 200 mM NaCl, 10 mM maltose, pH 7.4). Selected fractions were run on a 12% SDS–PAGE gel. The same protocol was followed for the purification of lysate containing only Ub-His-Trx5-CC C743S/C788S with amylose resin.

### Expression and purification of the TXNDC11 Trx5 domain

Competent BL21(DE3) cells were transformed with the plasmid pLE524 and plated on a LB agar plate containing kanamycin (50 μg ml^−1^). The next day, one colony was inoculated into 125 ml LB medium with kanamycin and the culture grown overnight at 37 °C. The overnight culture was diluted in LB with kanamycin to an optical density at 600 nm (OD_600_) of 0.1 and grown at 37 °C until the OD_600_ reached 0.75. Protein expression was induced by addition of 0.05 mM IPTG and the cells were cultivated at 20 °C overnight. Cells were harvested by centrifugation and cell pellets stored at −20 °C until lysis. Cell pellets were dissolved on ice in 20 ml lysis buffer (50 mM Na-phosphate, 300 mM NaCl, 10 mM imidazole, pH 8 containing protease inhibitor cocktail and 1 mM PMSF) per liter of starting culture. Cells were broken open upon incubation with 1 mg ml^−1^ lysozyme on ice for 1 h followed by sonication. The lysate was centrifuged for 1 h at 27,000 × *g* at 4 °C. The fusion protein was purified by incubation of the filtered supernatant with Ni-NTA beads (Qiagen) prewashed in Ni-NTA binding buffer (50 mM Na-Phosphate, 300 mM NaCl, 10 mM Imidazole, pH 8.0) for 1 h and 15 min at 4 °C on a rolling table. The beads and supernatant were then transferred to an empty column placed at 4 °C and the FT collected. The beads were washed with at least 25 column volumes (CVs) of Ni-NTA wash buffer (50 mM Na-phosphate, 300 mM NaCl, 20 mM Imidazole, pH 8.0) and 10 ml wash fractions were collected. Elution of bound Ub-His-Trx5 C743S was achieved by addition of 15 CVs of Ni-NTA elution buffer (50 mM Na-phosphate, 300 mM NaCl, 400 mM Imidazole, pH 8.0), and the protein visualized on a Coomassie-stained 15% reducing SDS–PAGE gel. Relevant fractions were pooled before cleavage of the Ub-His-tag, which was done by incubation of the protein pool with 1 mM EDTA, 10 mM β-mercaptoethanol and the sTEV protease (plasmid was a kind gift from Prof. V. Dötsch, Goethe University Frankfurt, Germany) in a ratio of 1:25 sTEV:Ub-His-Trx5 C743S at room temperature for 3 h. Next, the cleavage reaction solution was dialyzed into Ni-NTA binding buffer at 4 °C overnight. To bind the cleaved Ub-His-tag, the dialyzed protein pool was incubated with Ni-NTA beads as described above. In most cases, small amounts of Ub-His-tag were still present in the FT. In these cases, the FT was re-incubated with Ni-NTA beads to bind the remaining Ub-His-tag. Next, the FT was concentrated to *A*_280_=0.5 using an Amicon spin filter. To remove the remaining contaminants, the concentrated protein pool was centrifuged for 15 min at 16,100 × *g* at 4 °C and loaded on a Superdex75 (GE Healthcare) gel filtration column. The Trx5 C743S domain was eluted isocratically in Trx5 C743S storage buffer (50 mM Na-phosphate, 150 mM NaCl, pH 7.0). The eluted fractions were analyzed by SDS–PAGE and pooled based on purity. The concentration of Trx5 C743S was calculated from the absorbance at 280 nm (recorded on a Zeiss Specord S10 with Aspect Plus software) and a theoretical extinction coefficient for oxidized Trx5 C743S of 21095 M^−1 ^cm^−1^ (ref. 48). Matrix assisted laser desorption ionization time-of-flight MS (MALDI-TOF MS) was performed on a Bruker Autoflex mass spectrometer. Sample preparation was carried out according to the dried droplet method using α-cyano-4-hydroxycinnamic acid (HCCA) matrix. Trx5 C743S was diluted to 3.75 μM in 0.2% TFA and mixed 1:1 with HCCA matrix solution (HCCA in 1:2 Acetonitrile:0.1% TFA). The spectrometer was calibrated using a quadratic fit to a set of standard proteins (Protein calibration standard 14,000–20,000 Da (Bruker)).

### CD spectroscopy

Purified Trx5 C743S was concentrated to A_280_=0.5 (23 μM) and dialyzed into CD buffer (10 mM Na-phosphate, pH 7.0) overnight at 4 °C. Before the CD measurements, the dialyzed protein was centrifuged for 30 min at 4 °C at 16,100 × *g*. The spectrum of the reduced form of Trx5 C743S was recorded on a sample pre-incubated for 1 h at 4 °C and 2.5 h at room temperature. The spectra were recorded at 5 °C at a scan rate of 20 nm min^−1^ from 260 to 190 nm on a Jasco-J810 spectropolarimeter equipped with a Peltier temperature control device. The spectra were recorded as an accumulation of 15 scans. Upon recording, all data sets were subtracted the buffer baseline and noise-reduced with a fast Fourier transform filter in the Spectra Manager software. The resulting ellipticities were normalized to concentration and number of amino acids using the equation:


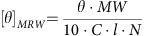


where *θ* is the ellipticity in degrees, *MW* is the molecular weight in g mol^−1^, *C* is the concentration of oxidized Trx5 C743S in g ml^−1^, *l* is the path length in cm and *N* is the number of amino acids in the sequence. For protein concentration determination, the absorbance at 280 nm was recorded immediately after the CD measurement of each sample on a UV-Vis spectrophotometer. The redox state of the proteins were quenched with 20% TCA and analyzed using the AMS shift assay (see below).

### AMS modification

In a final volume of 100 μl, 0.4 nmol purified Trx5 C743S was incubated with various reducing agents. Upon incubation, the redox state of the reactants was quenched with 20% TCA and protein precipitated for at least 30 min on ice. The precipitate was pelleted by centrifugation for 15 min at 4 °C at 16,100 × *g* and the supernatant discarded. Upon another centrifugation for 1 min, the remaining supernatant was discarded and 10 μl AMS buffer (0.4 M Tris–HCl pH 7.5, 1.6% SDS, 0.1 % bromocresolpurple, 15 mM AMS (4-acetamido-4′-maleimidylstilbene-2,2′-disulfonic acid; Fluka)) was added. The pH of the solution was titrated with 1.5 M Tris–HCl pH 8.8 until a colour shift was observed (usually 1 μl was enough). The pellets were dissolved by vortexing for 1 min and the AMS modification reaction carried out for 2 h at room temperature in the dark. Samples were then boiled in non-reducing loading buffer for 5 min, spun down and stored at −20 °C before analysis on 18% SDS–PAGE gels.

### Redox titration using lipoic acid

Stock solutions of 30 mg ml^−1^ dihydrolipoic acid (DHLA) (Sigma) and 50 mg ml^−1^ lipoic acid (LA) (Sigma) were prepared in 96% nitrogen-flushed ethanol and kept at −20 °C. Before setting up the experiment, the actual concentration of the DHLA stock was calculated using Lambert Beers law from the absorbance of Ellman's reagent (1 mM 5,5′-dithiobis-(2-nitrobenzoic acid), 50 mM K-phosphate, 0.1 mM EDTA, pH 7.3) at 412 nm and the extinction coefficient 14,150 M^−1 ^cm^−1^ (ref. 49). The amount of oxidized LA in the DHLA stock was determined from the absorbance at 333 nm and the extinction coefficient for oxidized LA in ethanol 170 M^−1 ^cm^−1^ (ref. 50). The actual concentration of the LA stock was determined similarly. Since the solubility of DHLA/LA is quite low in aqueous solution, the stock solutions were diluted in nitrogen-flushed 96% ethanol in appropriate concentrations, so when added to the redox titration samples, a final concentration of 11.5% ethanol was reached.

In the redox titration experiment, Trx5 C743S (4 μM) in nitrogen-flushed 50 mM Na-phosphate, 150 mM NaCl, 1 mM EDTA, pH 7.0 was incubated with theoretical DHLA/LA ratios ranging from 10 to 0.01 in a total volume of 100 μl. As air-oxidation of DHLA during the experiment would affect the resulting reduction potentials of the buffers, a set of blank samples identical to each protein sample (except that no protein was added) was prepared. After 16 h of incubation at 25 °C with shaking, the redox state of the protein samples was quenched by the addition of 20% TCA. The protein was precipitated on ice for at least 30 min, and the AMS modification protocol described above was followed. The blank samples were put on ice, and the actual DHLA and LA concentrations in each blank sample were determined spectrophotometrically using the methods described above. The only exception was that LA was measured at 330 nm using the extinction coefficient 127 M^−1 ^cm^−1^ for LA in phosphate buffer^51^. The reduction potential of the blank samples was calculated from the determined DHLA/LA ratios using the Nernst equation. A standard reduction potential of DHLA/LA of −290 mV was used^52^.

### Immunoblotting

Cells were lysed in 1% digitonin in TBS plus 10 mM iodoacetamide (IAA), 0.5 mM PMSF on ice for 30 min. Following centrifugation at 13,000 × *g* for 10 min, the post-nuclear supernatant was heated to 70 °C in SDS sample buffer for 10 min, separated by SDS–PAGE, and transferred to a PVDF membrane (Millipore). Membranes were blocked in 5% milk in PBS+0.2% Tween-20, probed with the indicated antibodies, and reactive bands visualized using West Pico or West Dura (Thermo Fisher Scientific). Uncropped gel images can be found in Supplementary Fig. 9.

### Co-immunoprecipitation and MS

For immunoprecipitation, digitonin lysates were pre-cleared with protein A and IgG-sepharose and incubated with primary antibody and protein A- or protein G-sepharose for 2 h at 4 °C. Following three washes in lysis buffer, samples were eluted in SDS sample buffer. For analysis by MS, immunoprecipitates were first resolved by SDS–PAGE, with each lane being cut into four slices for in-gel digestion. Tryptic peptides were analyzed by LC–MS/MS and the raw files processed in Proteome Discoverer 1.4. Data was searched using Sequest against the Human Uniprot database (downloaded 03/03/14, 68710 sequences).

### qRT-PCR

The RNeasy Plus kit (Qiagen) was used to extract RNA, which was then reverse transcribed using a poly(d)T primer and Super RT reverse transcriptase (HT Biotechnology). All reactions were performed using 4 ng of cDNA, 10 μl of SYBR green PCR mastermix (Life Technologies) and 0.2 μM forward and reverse primers in a final reaction volume of 25 μl. Samples were run on an ABI 7,500 Real Time PCR System (Applied Biosystems), with cycling parameters of 50 °C for 2 min and 95 °C for 5 min, followed by 40 cycles of 95 °C for 15 s and 58 °C for 1 min. Primer sequences can be found in Supplementary Data 4.

### Data availability

The authors declare that the data supporting the findings of this study are available within the article and its Supplementary Information files.

## Additional information

**How to cite this article:** Timms, R. T. *et al*. Genetic dissection of mammalian ERAD through comparative haploid and CRISPR forward genetic screens. *Nat. Commun.* 7:11786 doi: 10.1038/ncomms11786 (2016).

## Supplementary Material

Supplementary Information Supplementary Figures 1-9 and Supplementary References

Supplementary Data 1 Haploid gene-trap screen data. For each gene, the Bonferronicorrected Fisher's p-value is given together with the number of unique insertion sites identified in the gene. Gene-trap integrations are considered inactivating if they occur either in exons or in introns in the correct orientation for trapping to occur.

Supplementary Data 2 CRISPR screen data.

Supplementary Data 3 Analysis of TXNDC11 immunoprecipitates by mass spectrometry. Confirmed TXNDC11 binding partners are highlighted in green.

Supplementary Data 4 Oligonucleotide sequences.

## Figures and Tables

**Table 1:** Identification of TXNDC11 binding partners by mass spectrometry.

	Wild-type		TXNDC11 knockout	
	Peptides	Coverage (%)	Peptides	Coverage (%)
Protein disulphide-isomerase (PDI)	22	40	1	3.1
ER degradation-enhancing alpha-mannosidase-like protein 2 (EDEM2)	20	37	0	0
Glucosidase 2 subunit alpha (GANAB)	19	23	0	0
Thioredoxin domain-containing protein 11 (TXNDC11)	19	22	0	0
ER degradation-enhancing alpha-mannosidase-like protein 3 (EDEM3)	15	21	0	0
Glucosidase 2 subunit beta (GLU2B)	13	26	0	0
Thioredoxin domain-containing protein 5 (TXNDC5)	10	28	0	0
