# Supplementary material for: Genetic dissection of mammalian ERAD through comparative haploid and CRISPR forward genetic screens
Source: Nat Commun. 2016 Jun 10;7:11786. doi: 10.1038/ncomms11786 (PMC4906395; doi:10.1038/ncomms11786)
Supplement: Supplementary Information — Supplementary Figures 1-9 and Supplementary References [file ncomms11786-s1.pdf]

a

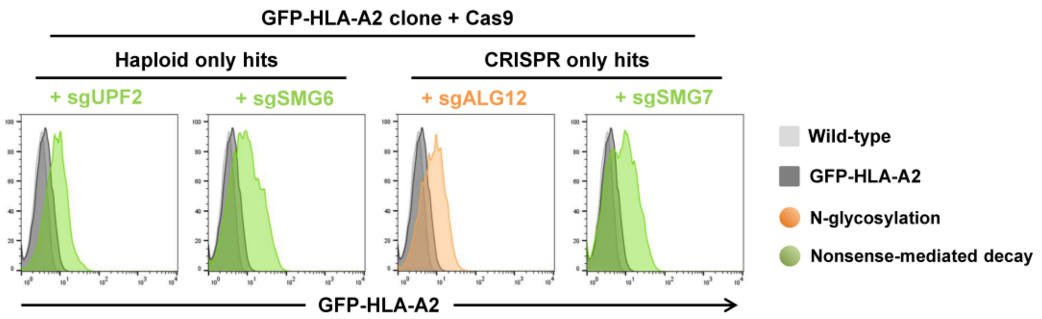

b

Gene	sgRNA_ID	sgRNA counts: Lib	S2
UPF2	HGLibA_60346	108	0
UPF2	HGLibA_60347	429	0
UPF2	HGLibA_60348	443	0
UPF2	HGLibB_52993	77	2435
UPF2	HGLibB_52994	167	12
UPF2	HGLibB_52995	335	262

Gene	sgRNA_ID	sgRNA counts: Lib	S2
SMG6	HGLibA_52991	107	256
SMG6	HGLibA_52992	72	8
SMG6	HGLibA_52993	87	0
SMG6	HGLibB_45648	41	0
SMG6	HGLibB_45649	14	11
SMG6	HGLibB_45650	180	0

c

			Unique integration sites detected			
		Bonferoni	Selected cells		Unselected library	
Rank	Gene	p-value	Inactivating	Total	Inactivating	Total
41	ALG12	0.011	9	10	3	7
1529	SMG7	727.2	8	9	25	49

d

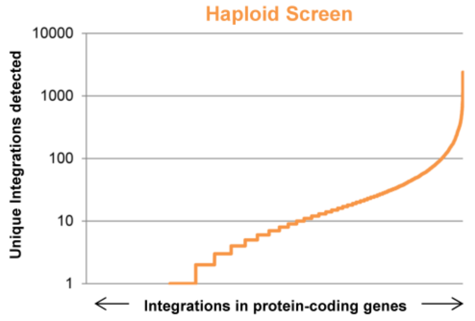

e

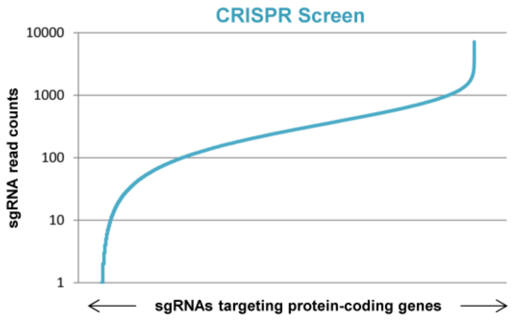

Supplementary Figure 1 | Investigating the genes identified differentially by the haploid gene-trap and CRISPR-mediated forward genetic screens. (**a**) Putative hits identified by only one of the screening approaches can be validated by individual CRISPR/Cas9-mediated gene disruption experiments. Ablation of *UPF2* and *SMG6*, identified only by the haploid gene-trap screen, and *ALG12* and *SMG7*, identified solely by the CRISPR screen, results in an increase in GFP-HLA-A2 reporter levels. (**b,c**) Illumina sequencing data relating to *UPF2*, *SMG6*, *ALG12* and *SMG7*. All six sgRNAs targeting *UPF2* and *SMG6* were detected in the unselected library from the CRISPR screen, but only a single sgRNA in each case was enriched in the selected population (b) (Lib, unselected control library; S2, selected cells after sort 2). In the haploid gene-trap screen *ALG12* fell just below our stringent cut-off for statistical significance; an increase in the number of inactivating insertions in *SMG7* was not observed in the selected cells (c). (**d,e**) Comparing the mutagenic coverage achieved using the two screening approaches. In each case, the mutagenic coverage across the 19,050 protein-coding genes targeted by the GeCKO v2 library in the unselected library sample was considered. Quantified are the number of unique integrations of the gene-trap vector detected (d) or the number of times each individual sgRNA was read (e). The CRISPR approach affords more even mutagenic coverage across the genome, as it is not subject to the integration bias of the retroviral mutagen. However, the gene-trap retrovirus is biased towards integration into actively expressed genes[1], and is capable of targeting all accessible chromatin rather than certain pre-designated genomic features.

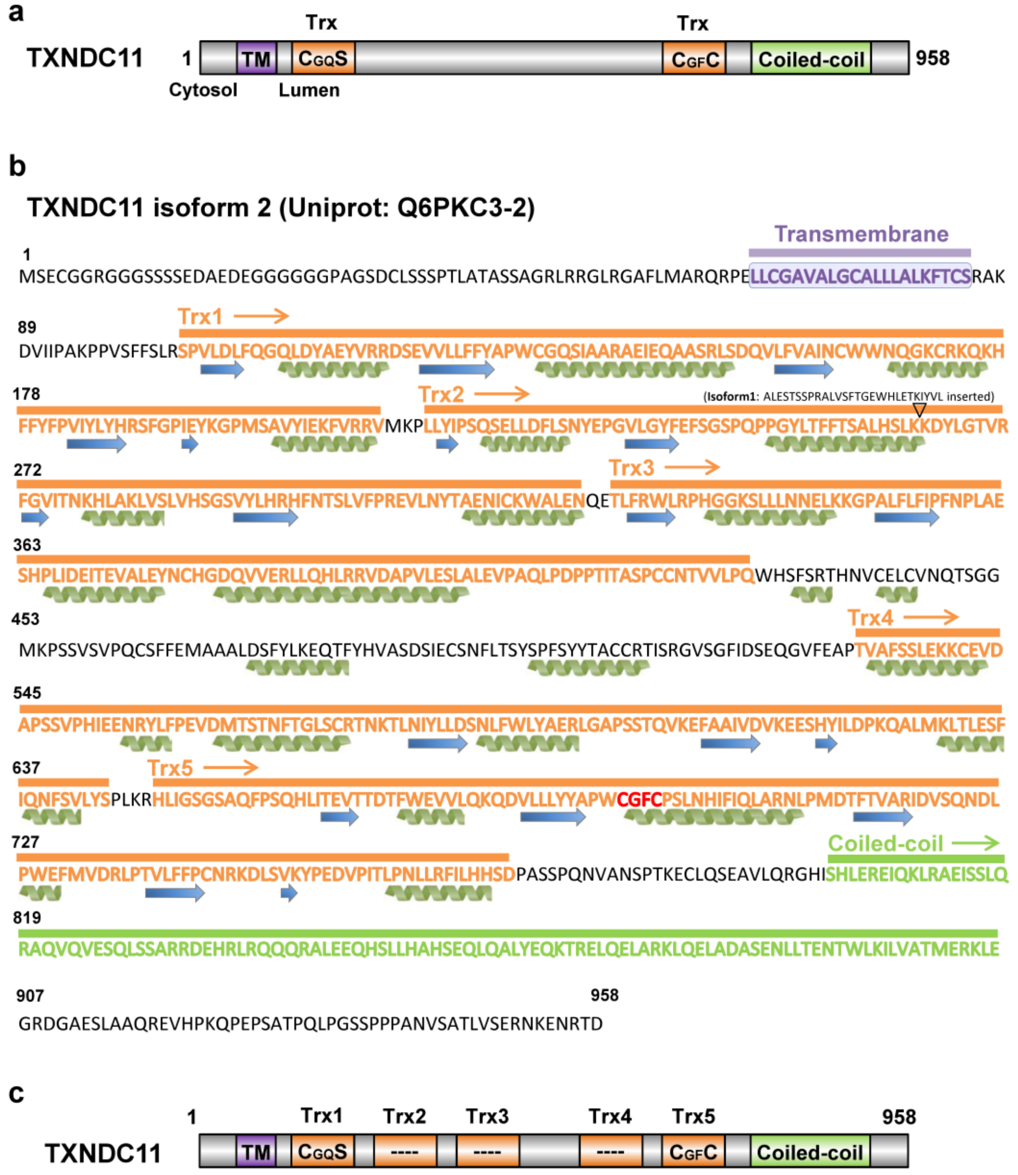

Supplementary Figure 2 | Identification of thioredoxin-like domains in TXNDC11. (**a**) The existing Uniprot annotation identifies TXNDC11 as a protein containing a short cytosolic N-terminus, a single transmembrane domain, two thioredoxin (Trx)-like domains in the luminal portion and a C-terminal coiled-coil domain. The last thioredoxin domain contains a canonical CXXC active site motif (CGFC), whereas the first Trx-like domain contains a CXXS motif (CGQS). (**b**) Homology modelling of the luminal portion of TXNDC11 predicts five thioredoxin-like domains. Using a combination of three homology modelling servers, iTasser[2], Phyre2[3] and Modeller[4], we identified an additional three probable Trx-like domains in TXNDC11. The Trx1, Trx2, Trx4 and Trx5 domains could be modelled in isolation, i.e. as separate entities, whereas the Trx3 domain was modelled by all three servers when using the region comprising Trx1-3 as the input sequence. Isoform 2 is depicted; Uniprot also identifies a TXNDC11 isoform 1, which is identical to isoform 2 apart from a 27 amino acid insertion into the Trx2 domain. Phyre2 and iTasser also provide some support for a sixth Trx-like domain located between Trx3 and Trx4, and, although this does not seem compatible with the secondary structure prediction, we cannot rule out the possibility of this additional domain. Secondary structure (green coil, α-helix; blue arrow, β-strand) predictions are manually assembled 'consensus' predictions based on the output from the three modelling servers. The approximate boundaries of the Trx domains are indicated by the orange bars, and the residues comprising the active-site CXXC motif of the Trx5 domain are highlighted in red. (**c**) Updated domain architecture prediction for TXNDC11.

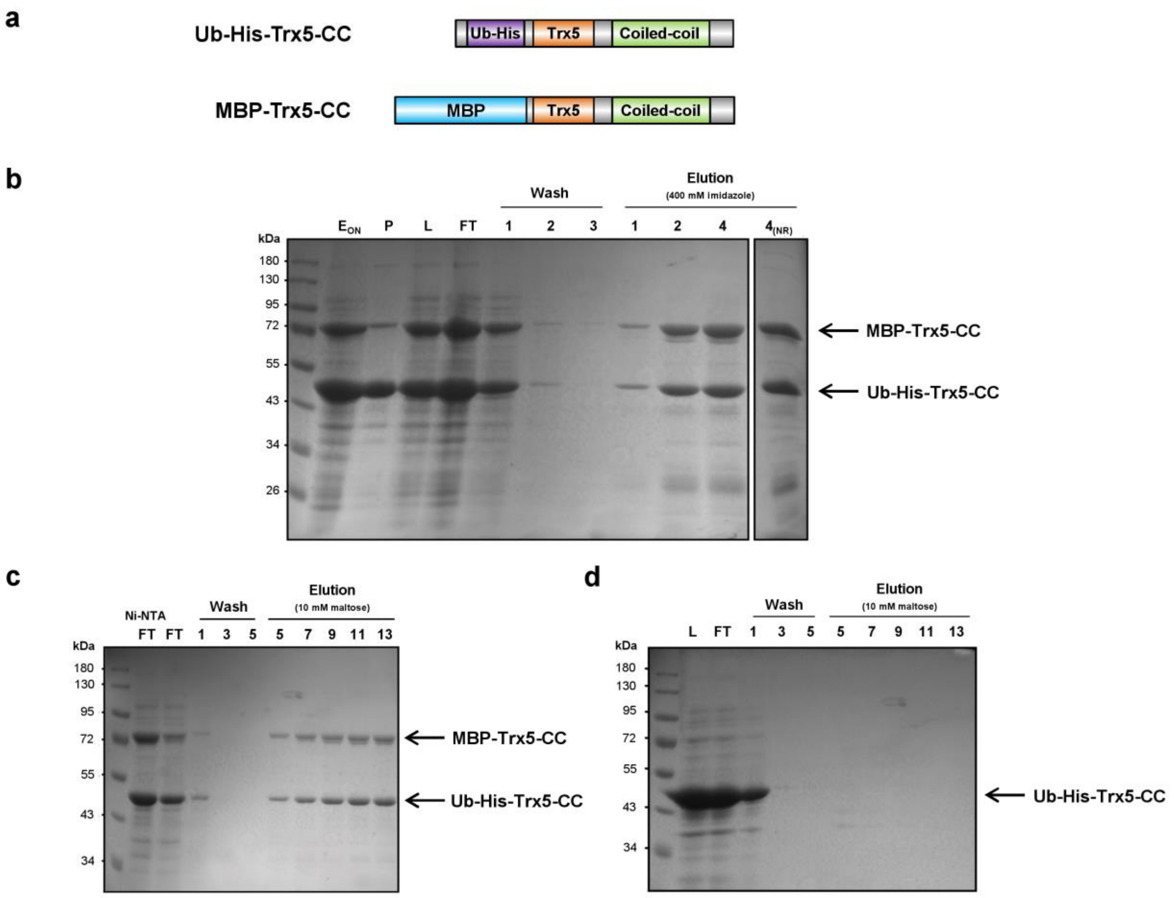

Supplementary Figure 3 | Oligomerisation of affinity-tagged TXNDC11 constructs containing the coiled-coil domain. (**a**) Schematic view of the constructs used. Two constructs encoding the Trx5 domain of TXNDC11 plus the coiled-coil (CC) region were co-expressed in *E. coli*: one contained N-terminal ubiquitin and His affinity tags (Ub-His-Trx5-CC), while the second contained a maltose binding protein (MBP) affinity tag (MBP-Trx5-CC). (**b**) Ni-NTA purification of co-expressed Ub-His-Trx5-CC and MBP-Trx5-CC. The MBP-tagged Trx5-CC construct co-elutes with the His-tagged Trx5-CC construct. (**c**) Elution profile of Ub-His-Trx5-CC and MBP-Trx5-CC upon rebinding of the flow-through (FT) from the Ni-NTA column to amylose resin. As in (b), the two constructs co-elute. (**d**) Elution profile of the Ub-His-Trx5-CC when expressed in isolation upon incubation with amylose resin. The His-tagged Trx5-CC construct does not bind the amylose resin in the absence of the MBP-tagged protein. (E_{ON}, total protein after 21 hours of expression; *P*, pellet after lysis; *L*, supernatant after lysis; *FT*, flow-through (unbound protein); numbers indicate washes or elution fraction. *NR*, non-reducing conditions)

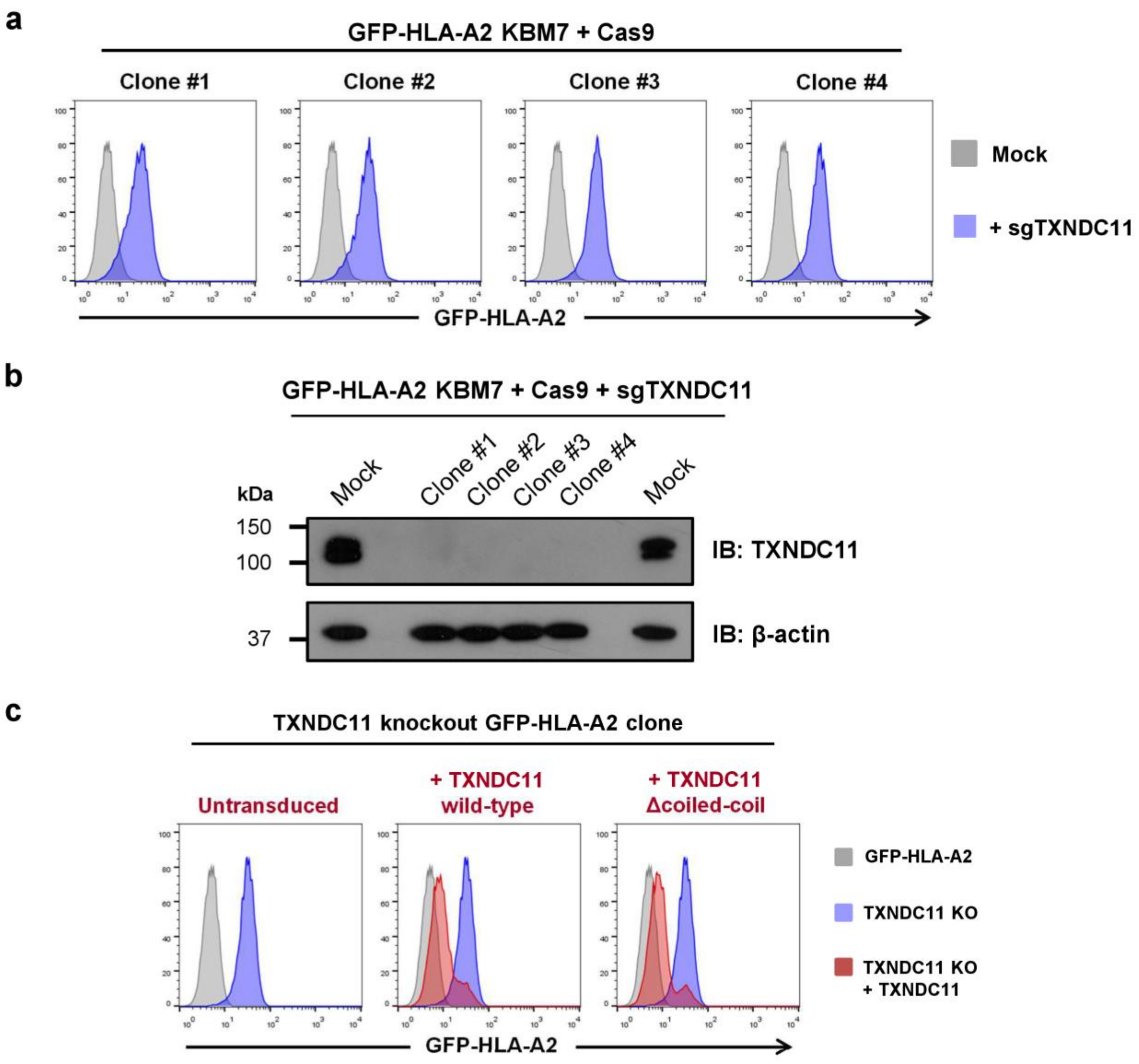

Supplementary Figure 4 | Genetic complementation of TXNDC11 knockout clones generated using CRISPR/Cas9 technology. (**a**) Isolation of TXNDC11 knockout clones. GFP-HLA-A2 KBM7 cells expressing Cas9 were transduced with two independent sgRNAs targeting TXNDC11, and GFP^{high} clones isolated. (**b**) Validation of the loss of TXNDC11 expression in these clones by immunoblot. (**c**) Genetic complementation of a TXNDC11 knockout clone upon exogenous expression of TXNDC11. Both wild-type TXNDC11 and a TXNDC11 mutant lacking the coiled-coil domain are able to rescue GFP-HLA-A2 degradation in TXNDC11 knockout cells.

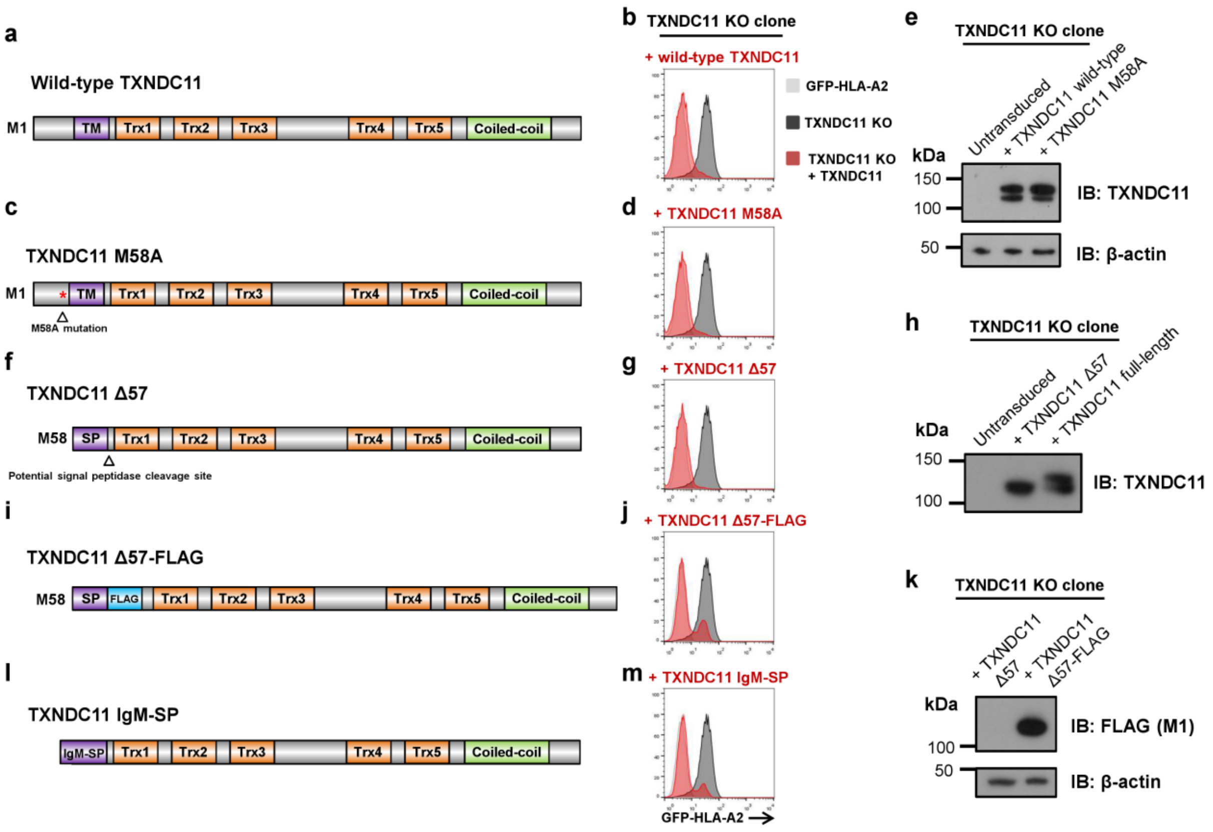

Supplementary Figure 5 | Investigating the origins of the twin isoforms of TXNDC11. Given that exogenous TXNDC11 migrated as a doublet similarly to the endogenous protein, we initially hypothesized that the twin isoforms of TXNDC11 could result from alternative translation initiation at Met^{58}, wherein the transmembrane domain (TM) could instead encode a signal peptide (SP). (**a-e**) To test this hypothesis we constructed a TXNDC11 mutant lacking the putative alternative translation initiation site at M58 (M58A) (c). This construct encoded a functional protein which could rescue the degradation of the GFP-HLA-A2 reporter in TXNDC11 knockout cells (d), but still migrated as a doublet on a polyacrylamide gel (e). Therefore, rather than alternative translation initiation, it is possible that partial signal peptidase cleavage is responsible for the twin TXNDC11 isoforms. (**f-k**) Translation initiation from Met^{58} produces a functional protein that is subject to signal peptide cleavage. To test whether translation initiation from Met^{58} would produce a functional protein, we constructed a TXNDC11 mutant lacking the first 57 amino acids (TXNDC11 Δ57) (f). This construct is functional (g) and the protein migrates as a single band on a polyacrylamide gel (h), alongside the faster migrating isoform of endogenous TXNDC11. To examine whether signal peptidase cleavage could occur, we next inserted a FLAG tag immediately downstream of the putative signal peptide (i), which, following signal peptidase cleavage, would be predicted to react with the M1 α-FLAG monoclonal antibody. The M1 antibody recognizes the FLAG epitope only when it is located at the free N-terminus of a fusion protein[5]. TXNDC11 Δ57-FLAG also encodes a functional protein (j), which is indeed M1-reactive (k). (**l,m**) TXNDC11 is functional when expressed downstream of an exogenous cleaved signal peptide. The TXNDC11 IgM-SP construct contains the signal peptide from murine immunoglobulin M in place of the first 87 residues of wild-type TXNDC11 (l). This construct can also restore GFP-HLA-A2 degradation in TXNDC11 knockout cells (m), showing that a soluble TXNDC11 protein retains the function of the wild-type protein.

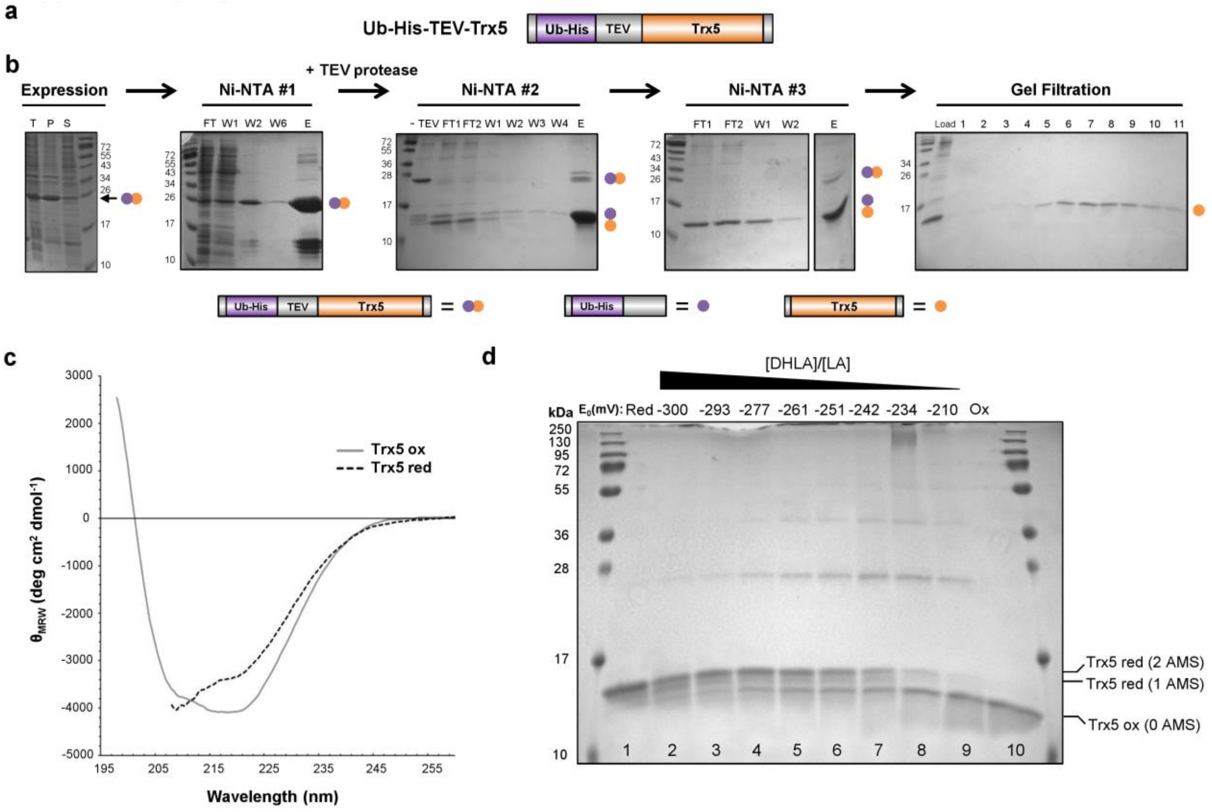

Supplementary Figure 6 | Redox properties of the TXNDC11 Trx5 domain. (**a,b**) Expression and purification of the TXNDC11 Trx5 domain in *E. coli*. A construct comprising the Trx5 domain of TXNDC11 (residues His^{650}-Asp^{772}) (a) was recombinantly expressed in *E.coli* as an Ubiquitin (Ub)-His-fusion protein and purified through Ni-NTA affinity chromatography, TEV protease cleavage of the Ub-His tag, and gel filtration (b). Mutation of a non-active site cysteine residue (C743S) was required to prevent the formation of disulfide-linked oligomers. (*T*, total protein after expression; *P*, pellet after lysis; *S*, supernatant after lysis; *FT*, flow-through (unbound protein); *W*, wash; *E*, elution. Numbers indicate different fractions.) (**c**) Analysis of Trx5 (C743S) by far-UV circular dichroism (CD) spectroscopy. The spectrum of the oxidized form (solid grey line) is similar to that of other Trx domains[6,7], representing a combination of β-strand (minimum at 215 nm) and α-helix secondary structure (minima at 208 nm and 222 nm), indicating that the protein is well folded. Upon reduction (dashed black line), the change in the spectrum suggests the protein undergoes a conformational change to a less well folded structure. Data collection from the reduced form was limited to the area between 208 and 260 nm, due to saturation of the high tension voltage from 190 nm to 207.5 nm caused by the reducing agent TCEP. (**d**) Redox titration of Trx5 (C743S) with lipoic acid (DHLA/LA). The full gel image from the experiment in Fig. 3g is shown. The alkylating agent 4-acetamido-4′-maleimidylstilbene-2,2′-disulfonic acid (AMS) reacts with free thiol groups, adding a mass of 490 Da per free thiol, thereby allowing the oxidized and reduced forms of the protein to be distinguished by a difference in mobility on polyacrylamide gels. Lipoic acid (DHLA/LA; reduction potential -290 mV[8]) was used for the titration, as the Trx5 domain was not efficiently reduced by the glutathione GSH/GSSG redox couple (reduction potential -240 mV) but was almost totally reduced at all ratios using DTT_{red}/DTT_{ox} as a redox couple (reduction potential -332 mV). Based on these data we estimate the reduction potential of the Trx5 domain to be between -234 mV and -242 mV. A more precise determination of this value is complicated by two factors. First, especially at

low DHLA/LA ratios, bands corresponding to dimers, trimers and higher oligomers appear. We speculate that these oligomers are disulfide-linked and that the less well-structured reduced form of Trx5 C743S could allow for the formation of these otherwise structurally unfavorable multimers. Second, a band corresponding to 1 AMS-modified Trx5 C743S was detected. This feature has previously been observed in shift assays[9], and can potentially be explained either by inefficient AMS modification or inaccessibility to AMS of one of the active-site cysteines of the reduced protein. However as we did not observe masses in the mass spectrum of the purified protein that could indicate hyperoxidation of a fraction of the cysteines, and as longer incubation times or greater amounts of the AMS did not affect the level of the 1 AMS band, we were not able to determine the exact nature and cause of the 1 AMS band.

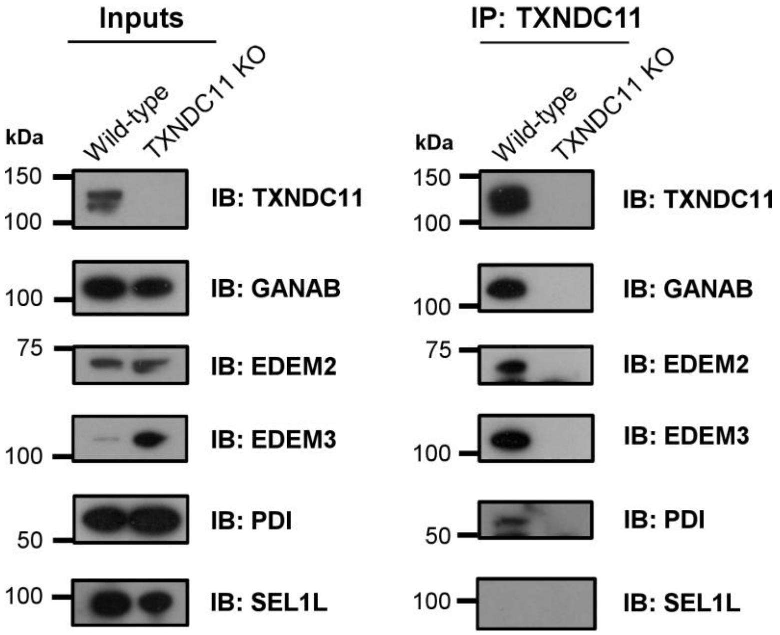

Supplementary Figure 7 | Immunoblot validation of the TXNDC11 binding partners identified by mass spectrometry. SEL1L, which was not identified as a TXNDC11-interacting protein, was used as a negative control.

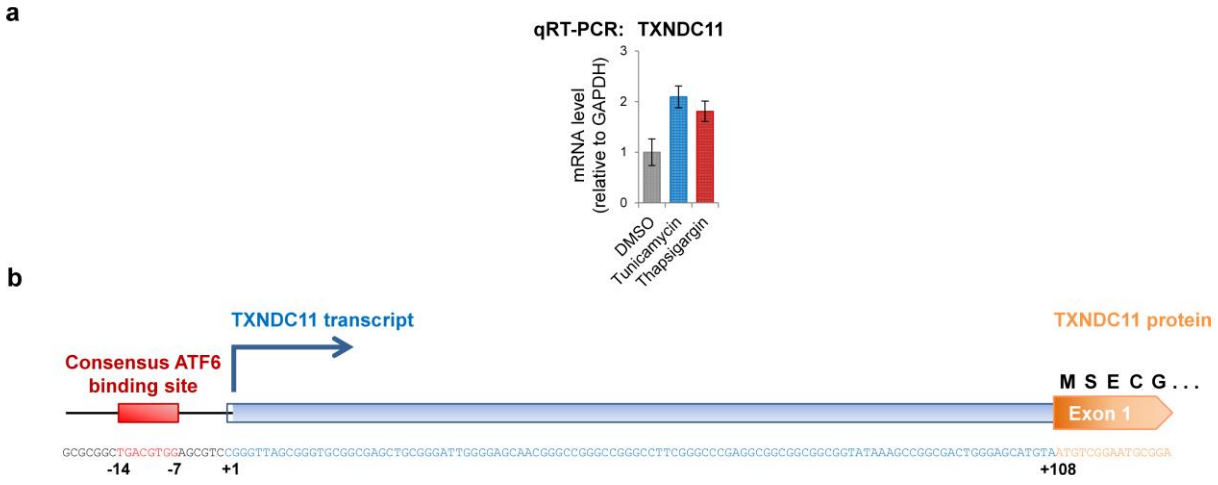

Supplementary Figure 8 | TXNDC11 expression increases upon ER stress. (**a**) Induction of TXNDC11 mRNA expression upon ER stress following treatment with tunicamycin and thapsigargin. Error bars represent the standard deviation of three technical replicates. (**b**) Identification of a potential ATF6 binding site in the TXNDC11 promoter. Visual inspection of the promoter region of the TXNDC11 gene identified a canonical ATF6 binding site motif[10] (TGACGTGG) immediately upstream of the transcriptional start site.

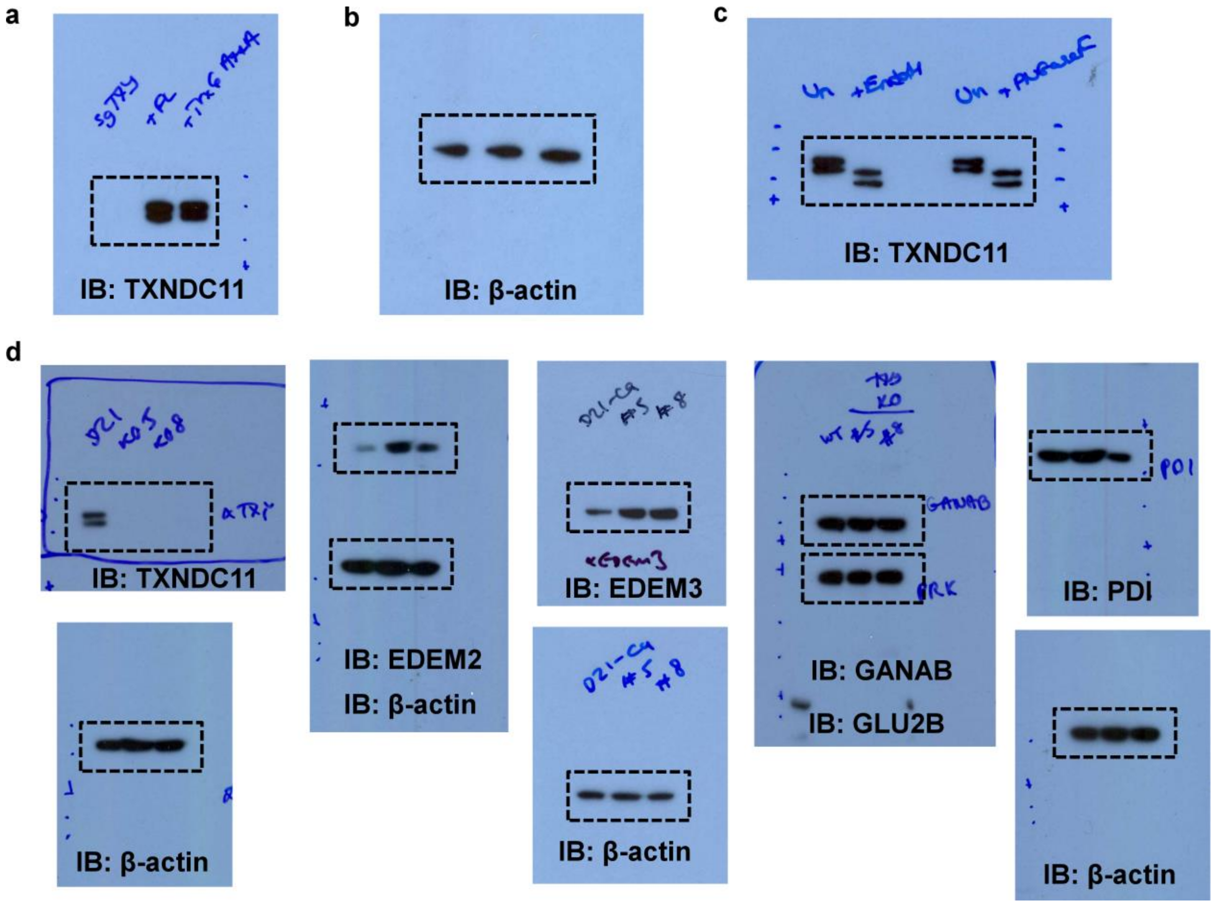

Supplementary Figure 9 | Full blots images. (**a-d**) Raw uncropped images of the blots shown in Fig. 3 and Fig. 4 are shown.

Supplementary References

1. Carette, J. E. *et al.* Global gene disruption in human cells to assign genes to phenotypes by deep sequencing. *Nat. Biotechnol.* **29,** 542–6 (2011).

2. Yang, J. *et al.* The I-TASSER Suite: protein structure and function prediction. *Nat. Methods* **12,** 7–8 (2014).

3. Kelley, L. A., Mezulis, S., Yates, C. M., Wass, M. N. & Sternberg, M. J. E. The Phyre2 web portal for protein modeling, prediction and analysis. *Nat. Protoc.* **10,** 845–858 (2015).

4. Webb, B. & Sali, A. Comparative Protein Structure Modeling Using MODELLER. *Curr. Protoc. Bioinformatics* **47,** 5.6.1–5.6.32 (2014).

5. Einhauer, A. & Jungbauer, A. The FLAG peptide, a versatile fusion tag for the purification of recombinant proteins. *J. Biochem. Biophys. Methods* **49,** 455–65 (2001).

6. Haugstetter, J., Blicher, T. & Ellgaard, L. Identification and characterization of a novel thioredoxin-related transmembrane protein of the endoplasmic reticulum. *J. Biol. Chem.* **280,** 8371–80 (2005).

7. Sugiura, Y. *et al.* Novel thioredoxin-related transmembrane protein TMX4 has reductase activity. *J. Biol. Chem.* **285,** 7135–42 (2010).

8. Lees, W. J. & Whitesides, G. M. Equilibrium constants for thiol-disulfide interchange reactions: a coherent, corrected set. *J. Org. Chem.* **58,** 642–647 (1993).

9. Hagiwara, M. *et al.* Structural basis of an ERAD pathway mediated by the ER-resident protein disulfide reductase ERdj5. *Mol. Cell* **41,** 432–44 (2011).

10. Wang, Y. *et al.* Activation of ATF6 and an ATF6 DNA binding site by the endoplasmic reticulum stress response. *J. Biol. Chem.* **275,** 27013–20 (2000).
